# Supplementary material for: An electrochemically responsive B–O dynamic bond to switch photoluminescence of boron-nitrogen-doped polyaromatics
Source: Nat Commun. 2024 Jun 17;15:5166. doi: 10.1038/s41467-024-48918-6 (PMC11183244; doi:10.1038/s41467-024-48918-6)
Supplement: Supplementary file 1 — Supplementary Information [file 41467_2024_48918_MOESM1_ESM.pdf]

Supplementary Information

An electrochemically responsive B–O dynamic bond to switch photoluminescence of boron-nitrogen-doped polyaromatics

Baige Yang[1], Yu-Mo Zhang*[,1], Chunyu Wang[1], Chang Gu[1], Chenglong Li*[,1], Hang Yin*[,2], Yan Yan[3], Guojian Yang[1], and Sean Xiao-An Zhang*[,1]

[1]State Key Lab of Supramolecular Structure and Materials, College of Chemistry, Jilin University, Changchun, 130012, P. R. China;

[2]Institute of Atomic and Molecular Physics, Jilin University, Changchun, 130012, P. R. China;

[3]College of Instrumentation & Electrical Engineering, Jilin University, Changchun, 130012, P. R. China.

*e-mail:

seanzhang@jlu.edu.cn (S.X.-A.Z.);

zhangyumo@jlu.edu.cn (Y.-M.Z.);

chenglongli@jlu.edu.cn (C.L.);

yinhang@jlu.edu.cn (H.Y.)

Contents

Supplementary Figures 3

Supplementary Tables 25

Supplementary Methods 34

Supplementary Method 1: Materials 34
Supplementary Method 2: Instrument characterization 34
Supplementary Method 3: Electrochemistry 35
Supplementary Method 4: Synthesis 36
Supplementary Method 5: Fabrication of the electrofluorochromic (EFC) devices 37
Supplementary Method 6: The calculation of association constant (K_a) 38

Supplementary Notes 40

Supplementary References 41

Supplementary Figures

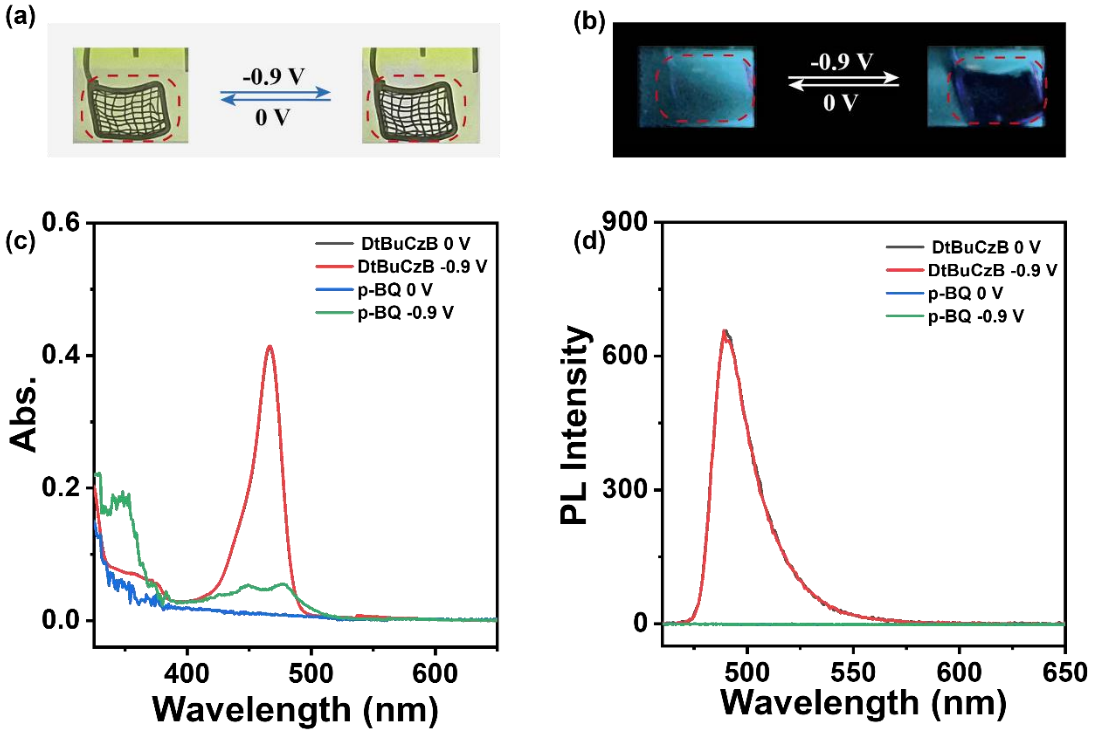

Supplementary Fig. 1. (a) and (b) actual picture of in-situ electrochemical cell with the mixture of *p*-BQ (1.0 × 10^{-3} mol L^{-1}) and DtBuCzB (1.0 × 10^{-4} mol L^{-1}) in THF with 1.0 × 10^{-1} mol L^{-1} tetrabutylammonium hexafluorophosphate ($TBAPF_6$) when the solutions were added 0 V and -0.9 V in-situ, respectively, ex = 440 nm. The spectra of absorption (c) and emission (d) spectra of alone *p*-BQ (1.0 × 10^{-3} mol L^{-1}) or DtBuCzB (1.0 × 10^{-4} mol L^{-1}) in THF with 1.0 × 10^{-1} mol L^{-1} $TBAPF_6$ when the solutions were added 0 V and –0.9 V in-situ, ex = 440 nm. Note: The black lines were completely covered by the red lines.

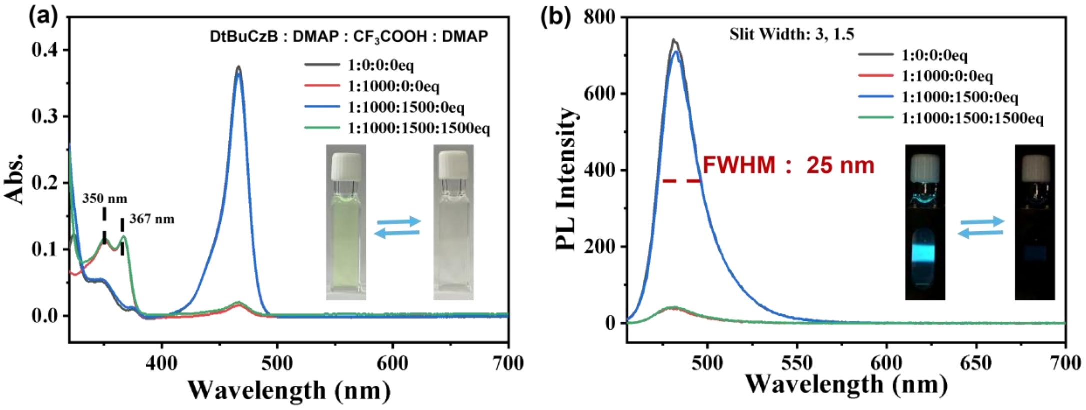

Supplementary Fig. 2. (a) Reversible absorption and (b) emission spectra of DtBuCzB (1.0×10^{-5} mol L^{-1}) in THF with the addition of DMAP (1.0×10^{-2} mol L^{-1}) and CF_3COOH (1.5×10^{-2} mol L^{-1}), ex = 440 nm.

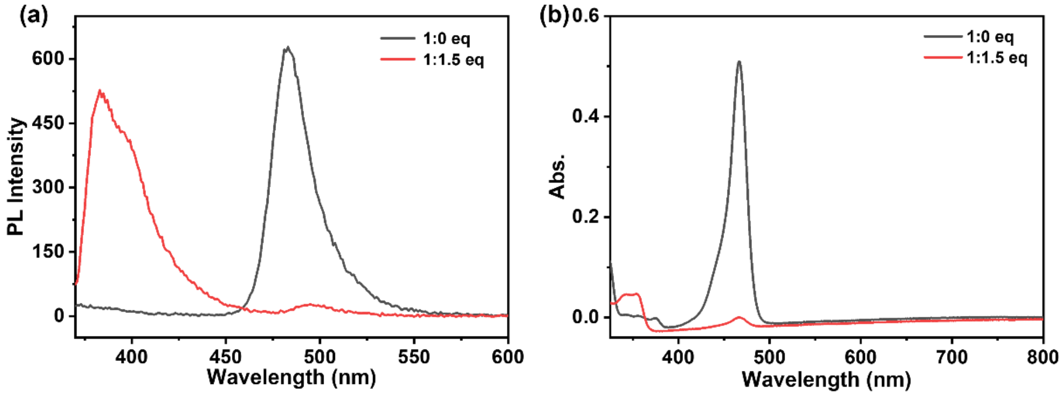

Supplementary Fig. 3. (a) Emission and (b) absorption spectra of DtBuCzB (1.0×10^{-5} mol L^{-1}) in THF with adding sodium phenolate (1.5×10^{-5} mol L^{-1}), ex = 350 nm.

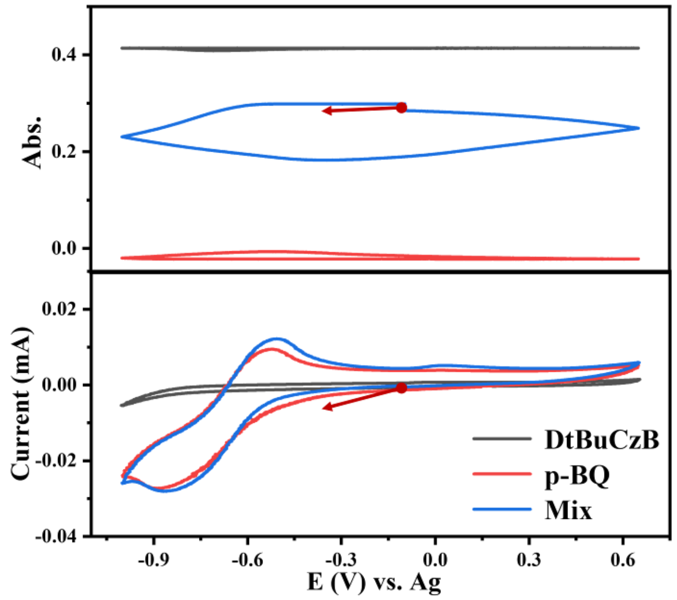

Supplementary Fig. 4. Changes in absorption spectra at 467 nm (top), during cyclic voltammograms (CVs, bottom) in situ of DtBuCzB (1.0×10^{-4} mol L^{-1}), *p*-BQ (1×10^{-3} mol L^{-1}), and the mixture (mix) of DtBuCzB and *p*-BQ (1.0×10^{-4} mol L^{-1} and 1.0×10^{-3} mol L^{-1}) in THF with 1.0×10^{-1} mol L^{-1} $TBAPF_6$.

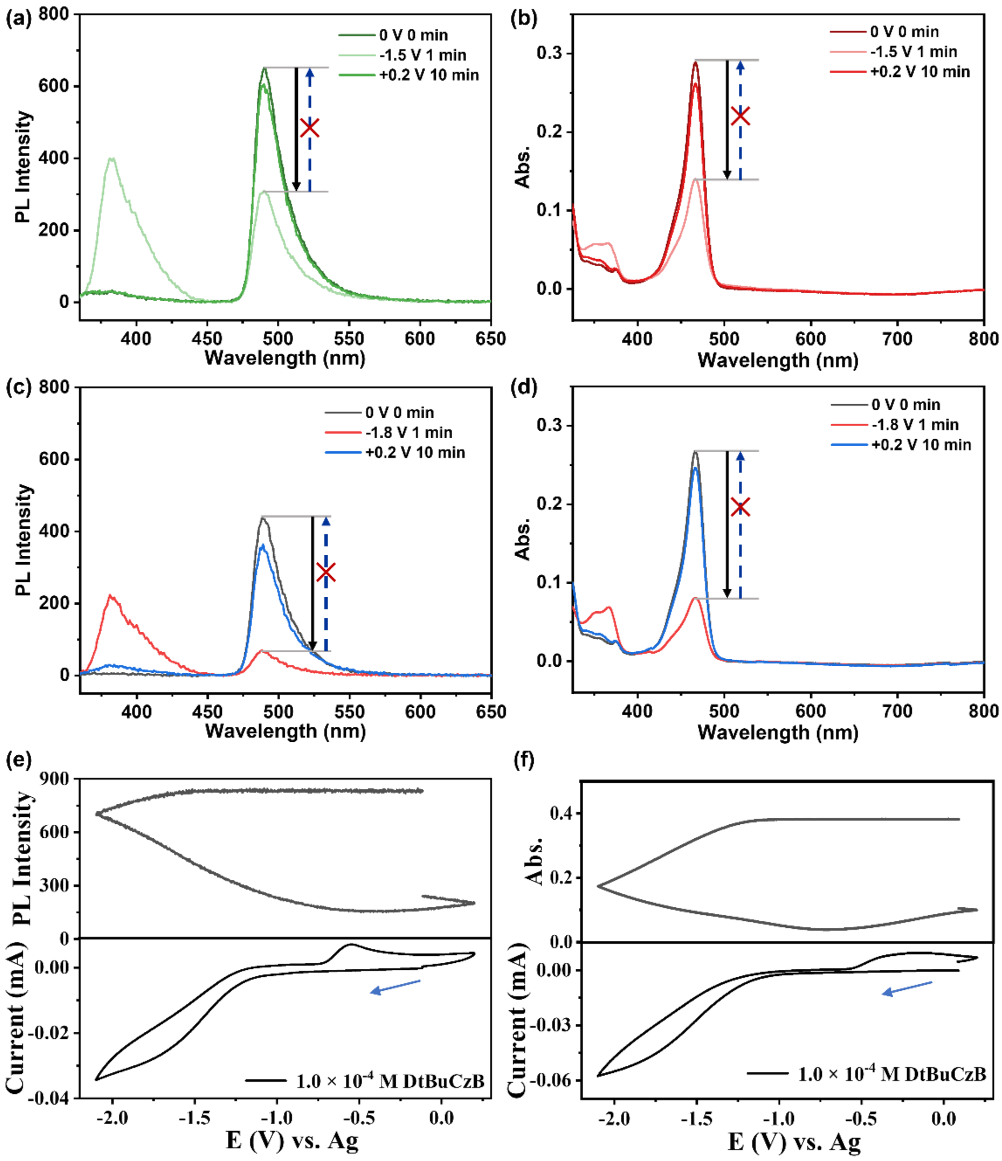

Supplementary Fig. 5 The spectra of (a, c) emission and (b, d) absorption spectra of alone DtBuCzB (1.0×10^{-4} mol L^{-1}) in THF with 1.0×10^{-1} mol L^{-1} $TBAPF_6$ when the solutions were added 0 V, –1.5 V/–1.8 V and +0.2 V in-situ, ex = 350 nm. Changes in (e) emission at 488 nm (top) and (f) absorption spectra at 467 nm (top), during cyclic voltammograms (CVs, bottom) in situ of DtBuCzB (1.0×10^{-4} mol L^{-1}) in THF with 1.0×10^{-1} mol L^{-1} $TBAPF_6$, ex = 440 nm.

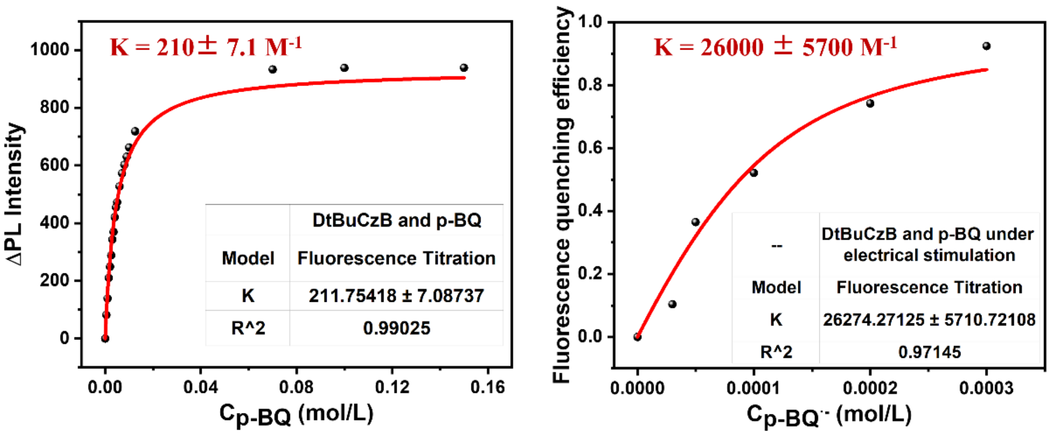

Supplementary Fig. 6. The non-linear curve-fitting (fluorescence titrations) for the complexation of (a) DtBuCzB (1.0×10^{-5} mol L^{-1}) and *p*-BQ without electrochemical stimulation, and (b) DtBuCzB (1.0×10^{-5} mol L^{-1}) and *p*-BQ under electrochemical stimulation in THF at 298 K.

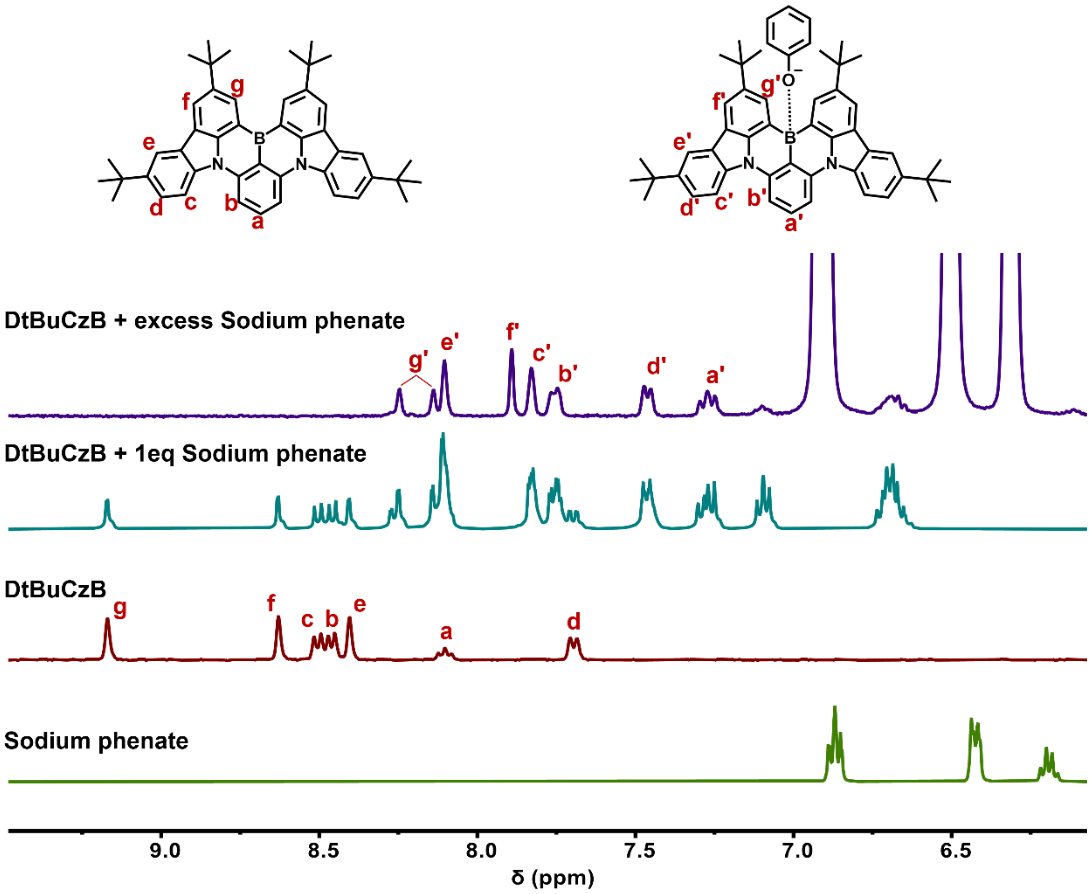

Supplementary Fig. 7. ^{1}H-NMR spectra of DtBuCzB, Sodium phenolate and the mixture of DtBuCzB and different equivalent Sodium phenolate in THF-d_8 recorded at 400 MHz at room temperature.

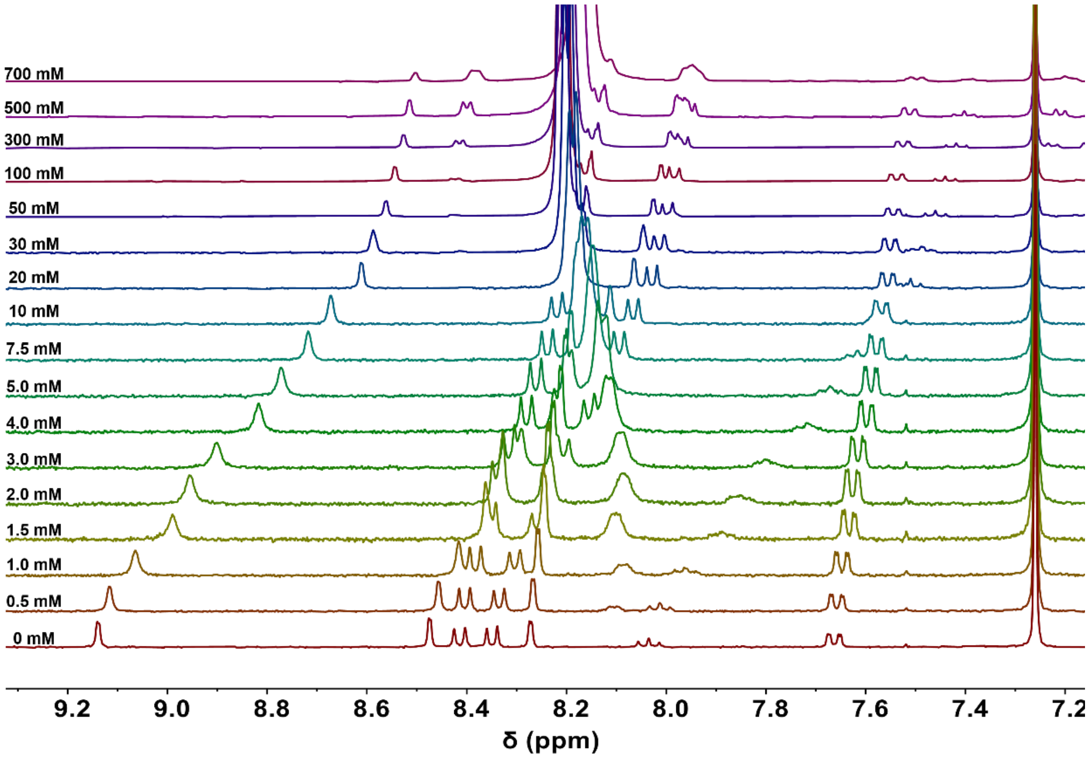

Supplementary Fig. 8. ^{1}H-NMR spectra (400 MHz, $CDCl_3$, 298 K) of DtBuCzB at a constant concentration of 1.25×10^{-3} mol L^{-1} by the addition of DMAP with different concentration.

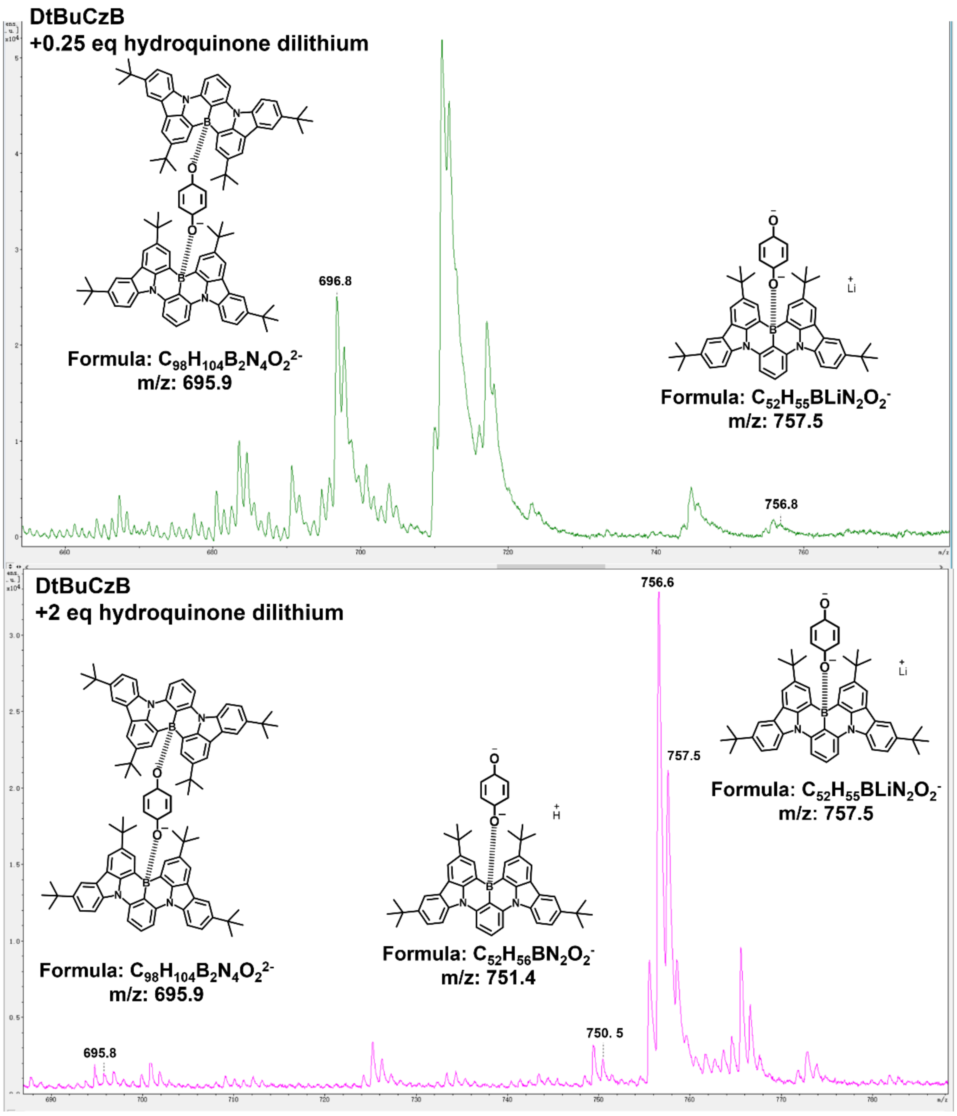

Supplementary Fig. 9. Mass spectrometry of the mixtures of DtBuCzB and different equivalent hydroquinone di-lithium in THF recorded at room temperature.

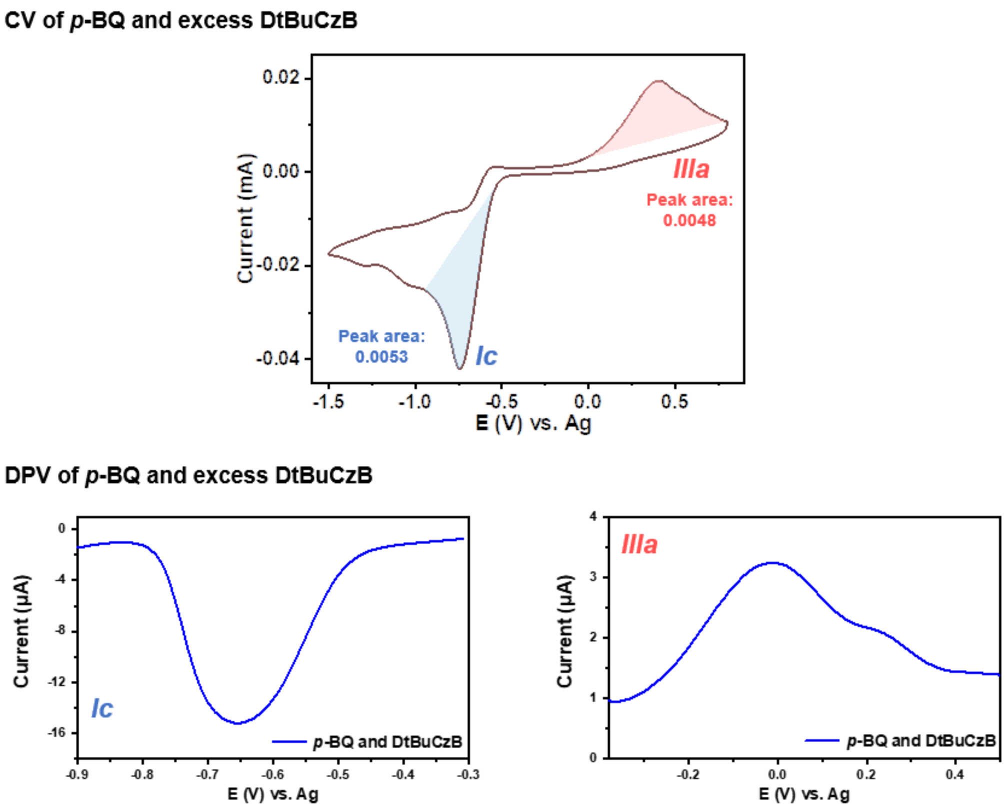

Supplementary Fig. 10. (a) Cyclic voltammogram of mixture of *p*-BQ (1.0×10^{-3} mol L^{-1}) with excess DtBuCzB in THF with 1.0×10^{-1} mol L^{-1} $TBAPF_6$, and the peak area of peak Ic and IIIa. The scan rate is 100 mV/s. Differential pulse voltammograms for (b) reduction peak Ic and (c) oxidation peak IIIc of mixture of *p*-BQ (1.0×10^{-3} mol L^{-1}) with excess DtBuCzB in THF with 1.0×10^{-1} mol L^{-1} $TBAPF_6$. Pulse amplitude of 50 mV, a pulse width of 50 ms, and scan rate: 25 mV/s.

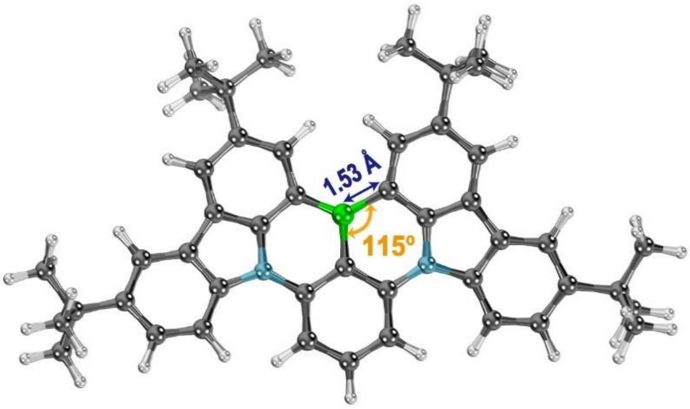

Supplementary Fig. 11. Optimized molecular structure, bond length and bond angle of DtBuCzB.

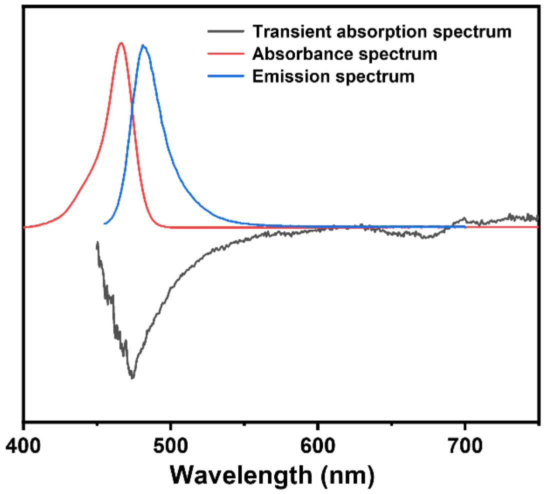

Supplementary Fig. 12. Comparison of steady-state absorption, emission and transient absorption spectra of DtBuCzB.

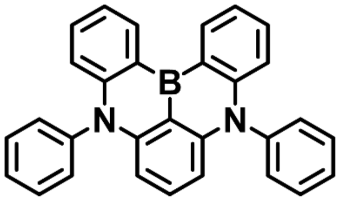

DABNA

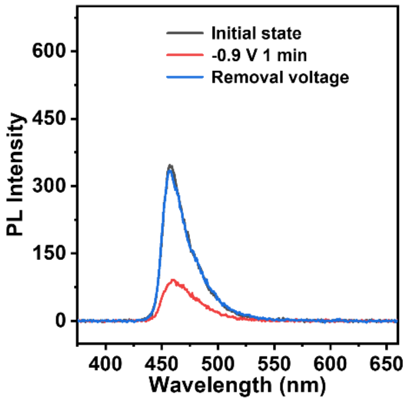

Supplementary Fig. 13. The emission of the mixture of *p*-BQ (1.0×10^{-3} mol L^{-1}) and DABNA (1.0×10^{-4} mol L^{-1}) in THF with 1.0×10^{-1} mol L^{-1} tetrabutylammonium hexafluorophosphate ($TBAPF_6$) before and after electrochemical stimulation.

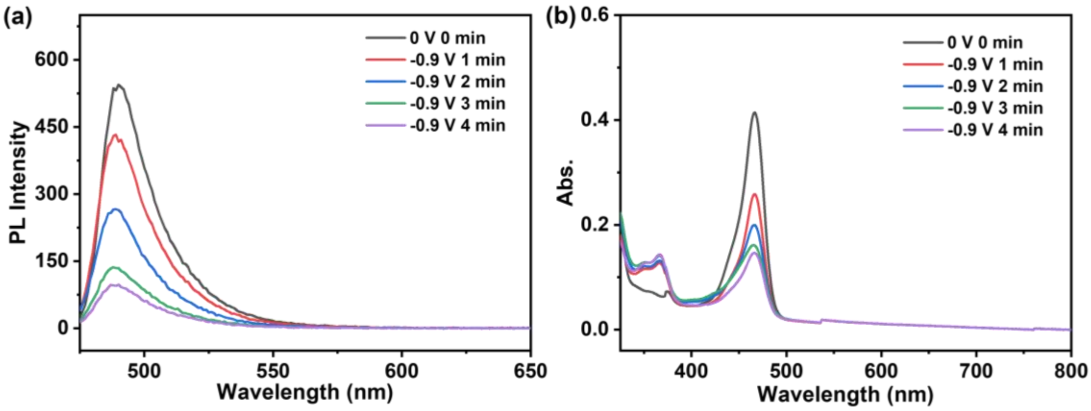

Supplementary Fig. 14. The emission (a) and absorption spectra (b) of the mixture of *p*-BQ (1.0×10^{-3} mol L^{-1}) and DtBuCzB (1.0×10^{-4} mol L^{-1}) in THF with 1.0×10^{-1} mol L^{-1} $TBAPF_6$ when the solutions were added –0.9 V in situ with different time.

The experimental results showed that the concentration of *p*-BQ can affect the coordination effect between DtBuCzB and *p*-BQ. As shown in **Supplementary Fig. 14**, the concentration of benzoquinone anions increased when a fixed voltage was applied to the mixed system, along with more obvious changes in photophysical properties in the system. Therefore, it can be concluded that the coordination effect was related to concentration.

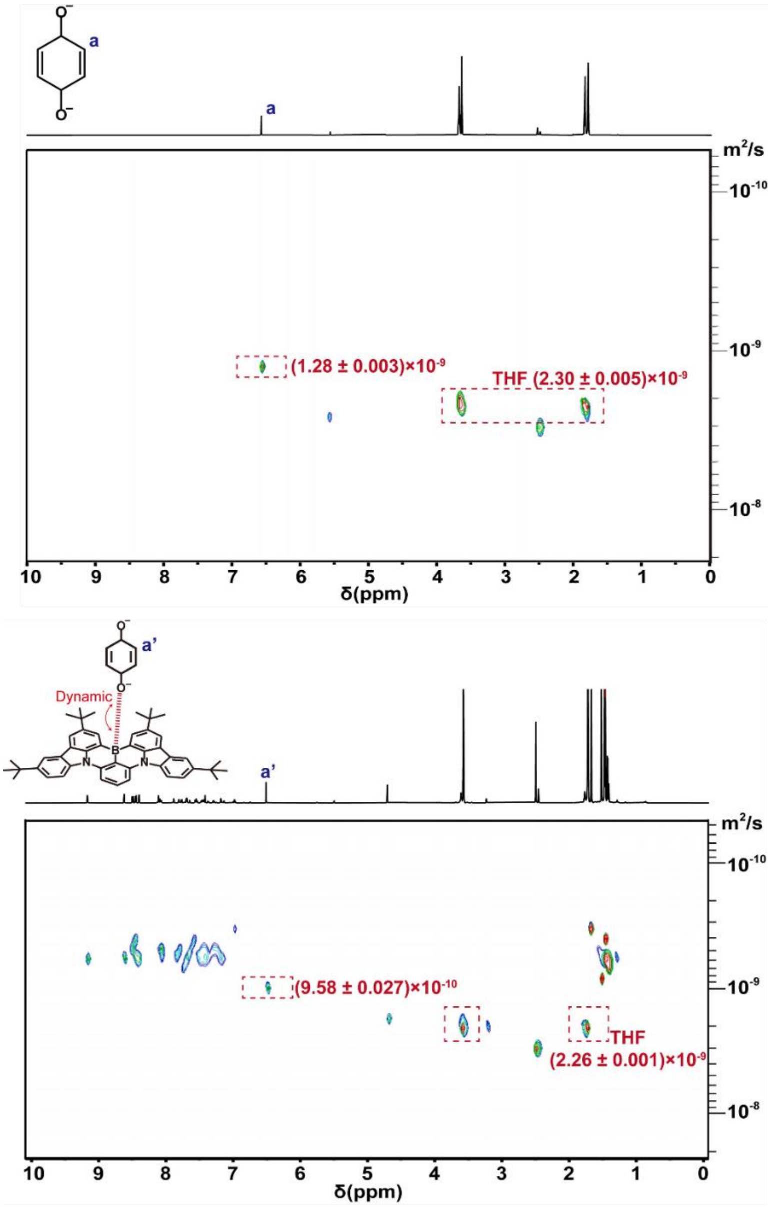

Supplementary Fig. 15 Diffusion ordered spectroscopy (DOSY, 600 MHz, *THF-d*$_8$) of free hydroquinone di-lithium, and the mixtures of DtBuCzB and 0.5 eq. hydroquinone di-lithium recorded at room temperature.

The representative ^{1}H-DOSY NMR spectra of the mixture were shown in **Supplementary Fig. 15**. It can be found that the diffusion coefficient of hydroquinone di-anion in the mixed system with DtBuCzB was lower than the corresponding value of the original free hydroquinone di-anion. These changes were directly related to the formation of dynamic interactions in this system.[S1-S2]

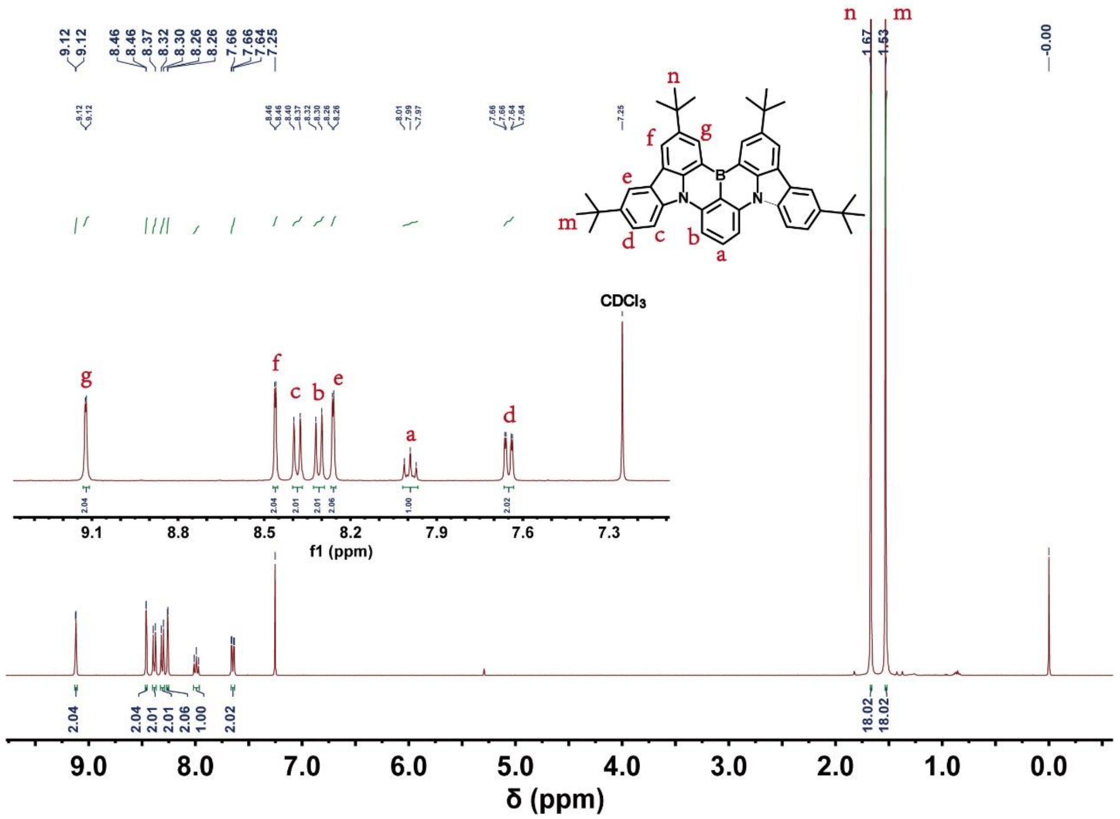

Supplementary Fig. 16. ^{1}H-NMR spectra of DtBuCzB in $CDCl_3$ recorded at 400 MHz at room temperature.

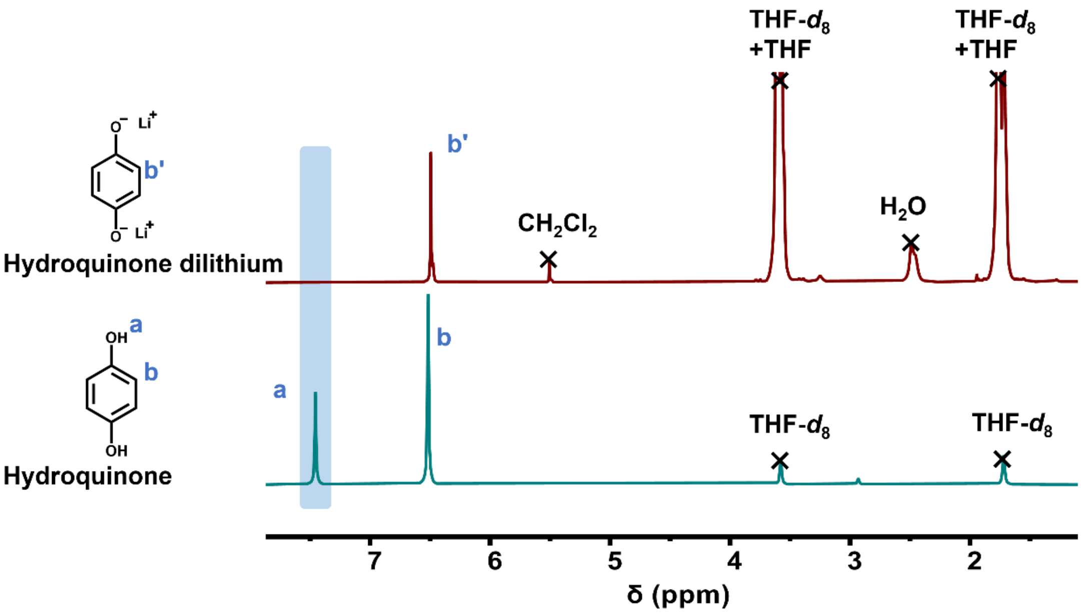

Supplementary Fig. 17. ^{1}H-NMR spectra of hydroquinone and the product (hydroquinone di-lithium) from hydroquinone and n-butyllithium in THF-d_8 recorded at 400 MHz at room temperature.

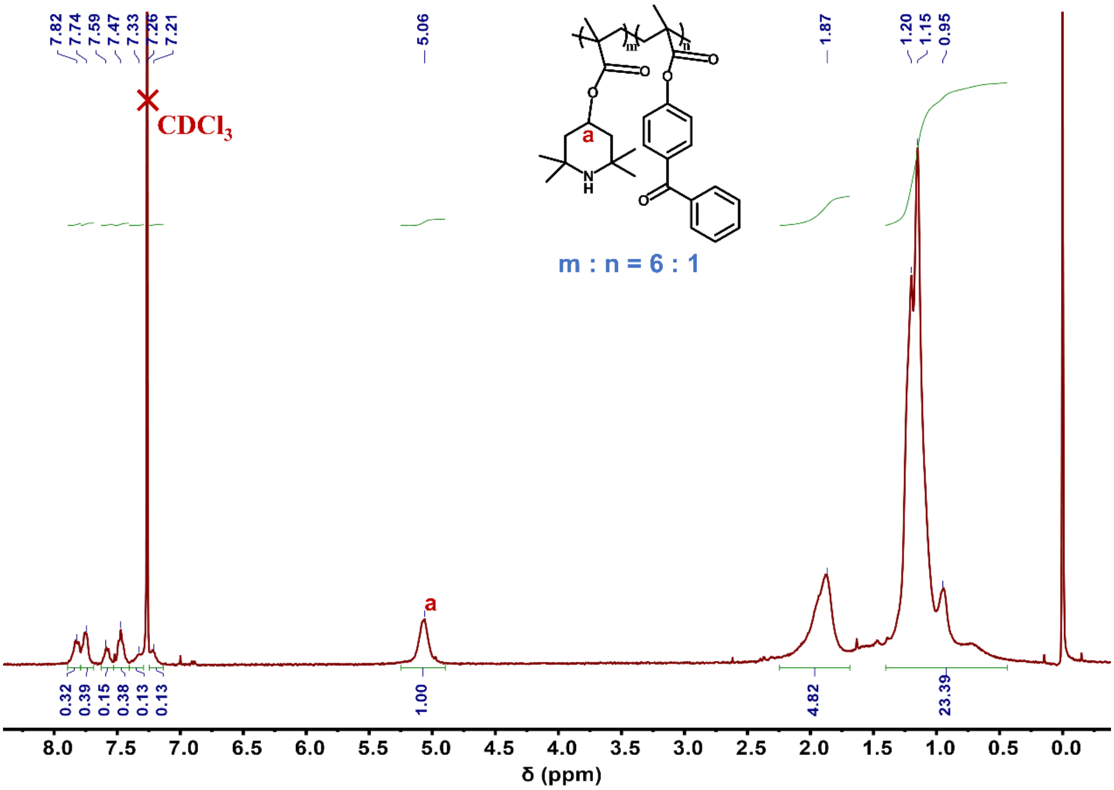

Supplementary Fig. 18. ^{1}H-NMR spectra of PTMPM-co-BP in $CDCl_3$ recorded at 400 MHz at room temperature. The ratio of the total aromatic protons (1.5, 9 H per unit) to the peak at 5.0 ppm, corresponding to the piperidine moieties (1, 1 H per unit), can be used to determine that m:n = 6:1.

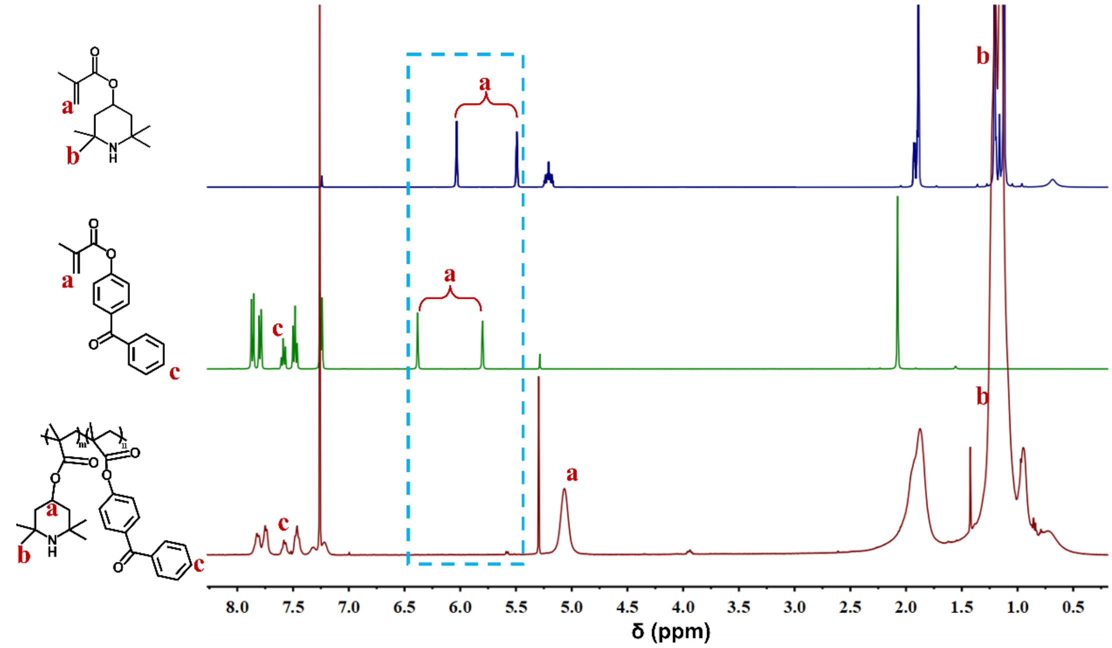

Supplementary Fig. 19. ^{1}H-NMR spectra of PTMPM-co-BP, BPMA and MTMP in $CDCl_3$ recorded at 400 MHz at room temperature.

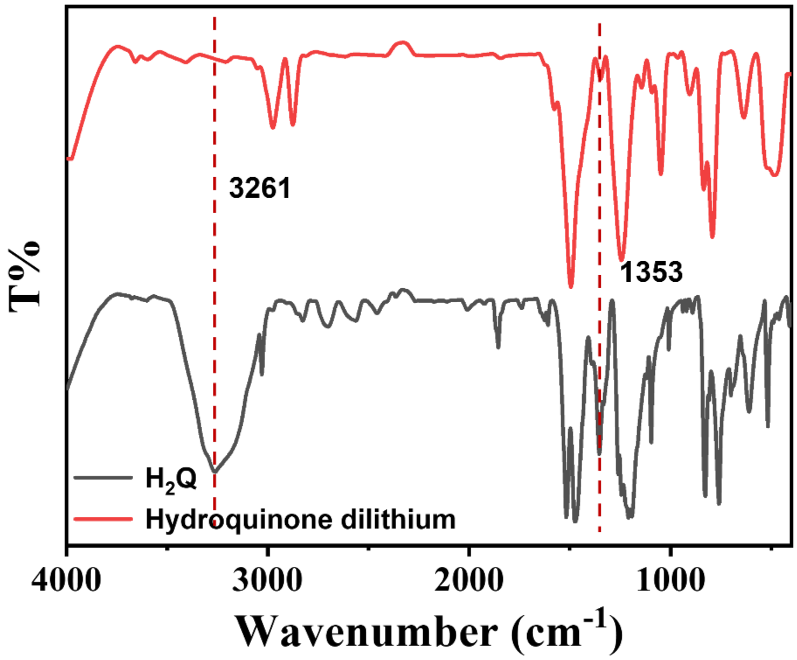

Supplementary Fig. 20. FT-IR spectra of hydroquinone (H_2Q) and hydroquinone di-lithium.

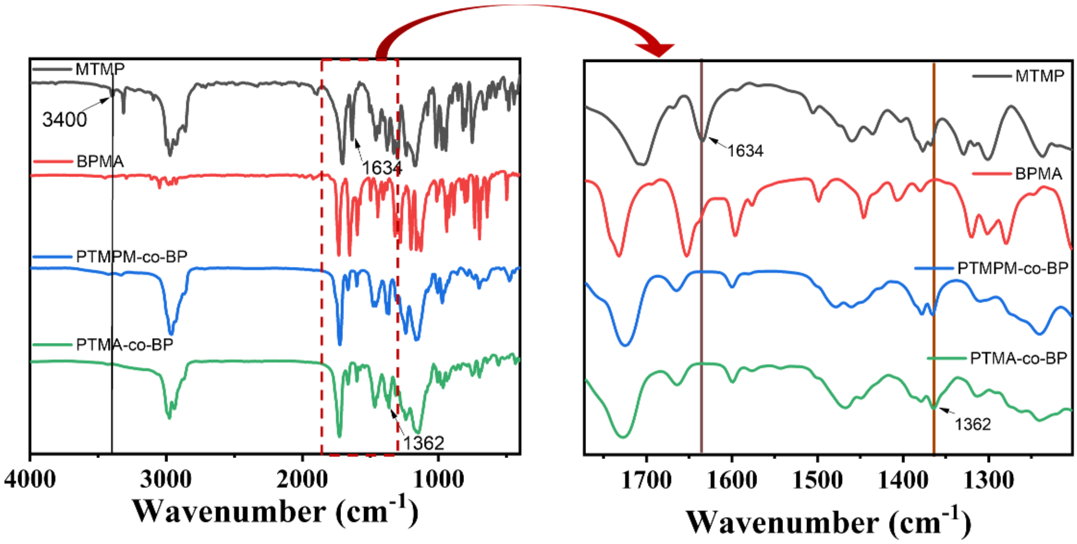

Supplementary Fig. 21. FT-IR spectra of PTMA-co-BP, PTMPM-co-BP, BPMA and MTMP.

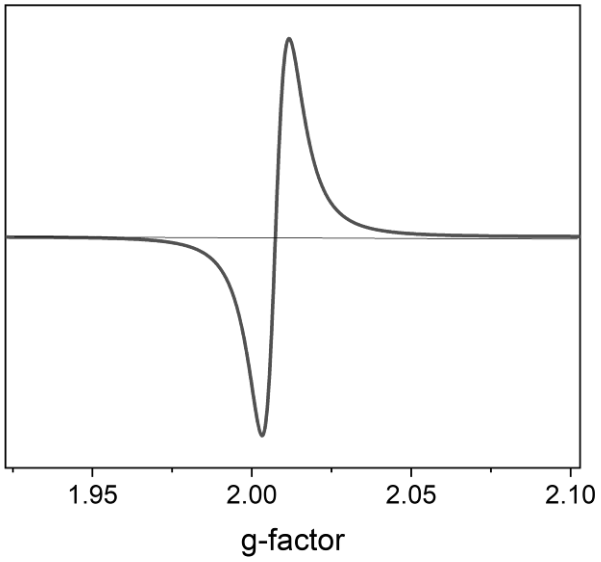

Supplementary Fig. 22. Electron paramagnetic resonance (EPR) spectrum of PTMA-co-BP recorded under the condition of FrequencyMon was 9.417976 GHz, BridgeCalib was 61.8, Power was 0.06325 mW, and PowerAtten was 35.0 dB in solid phase at room temperature.

Supplementary Tables

Supplementary Table 1. Experimental and theoretical photophysical properties of compounds that may be present in the system.

Compound	λ_{abs}[a)]/nm *(f)*	λ_{abs}[b)]/nm	λ_{ex}[a)]/nm *(f)*	λ_{ex}[b)]/nm
**DtBuCzB**	461 *(0.55572)*	467	491 *(0.59515)*	488
**Complex**[c)]	-	350, 367		
	372 *(0.15967)*			
**[(DtBuCzB)$_2$·*p*-BQ]$^{2-}$**	376 *(0.05845)*			
	391 *(0.06464)*			
	343 *(0.11629)*			
**[DtBuCzB·*p*-BQ]$^{2-}$**	362 *(0.03073)*			
	370 *(0.09653)*			
	647 *(0.01184)*			
**[(DtBuCzB)$_2$·*p*-BQ]$^{\bullet-}$**	926 *(0.00310)*			
	2013 *(0.00524)*			
	417 *(0.09864)*			
**[DtBuCzB·*p*-BQ]$^{\bullet-}$**	423 *(0.06608)*			
	460 *(0.02114)*			

[a)]Theoretical photophysical properties of compounds; [b)]Experimental photophysical properties of compounds; [c)]the complex was produced by in-situ electric stimulation of a mixture of DtBuCzB and *p*-BQ.

Supplementary Table 2. The summary of physical properties of DtBuCzB (sp^2-hybrid), **DtBuCzB·$OC_6H_5^-$** (sp^3-hybrid), and **DtBuCzB·$O_2C_6H_4^{2-}$** (sp^3-hybrid) in solution.

**Solution**	$\Phi_{PL,\ ex=440\ nm}$	$\Phi_{PL,\ ex=340\ nm}$	τ_p [ns]
**DtBuCzB**	91.49% (488 nm)	-	5.25
**DtBuCzB·$OC_6H_5^-$**	0.35% (488 nm)	21.12% (368 nm)	3.85
**DtBuCzB·$O_2C_6H_4^{2-}$**	0.18% (488 nm)	35.26% (366 nm)	3.70

Supplementary Table 3. The summary of basic performance of EFC device.

	λ_{em} [nm]	Φ_{PL} [a)]	Φ_{PL} [b)]	Contrast	ŋ	Switching time [s]
**EFC Device**	490	16.7%	1.0%	10.2	90%	t_{on}: 0.4<br>t_{off}: 0.8

[a)] Fluorescence quantum yield of initial device. [b)] Fluorescence quantum yield of device after electrochemical stimulation.

Supplementary Table 4. The absorption spectrum via transition electric dipole moments of DtBuCzB.

State	Energy [cm^{-1}]	Wavelength [nm]	fosc	T2 [au**2]	TX [au]	TY [au]	TZ [au]
1	21704	460.7	0.55572	8.42932	–2.90127	–0.00004	–0.10945
2	26853.4	372.4	0.00381	0.04671	–0.00001	0.21613	0.00001
3	27007.5	370.3	0.007307	0.08907	0.2213	0	–0.20023

Supplementary Table 5. The emission spectrum via transition electric dipole moments of DtBuCzB.

State	Energy [cm^{-1}]	Wavelength [nm]	fosc	T2 [au**2]	TX [au]	TY [au]	TZ [au]
1	20383.6	490.6	0.595146	9.61213	3.09768	0.00004	0.12856

Supplementary Table 6. The absorption spectrum via transition electric dipole moments of $[(DtBuCzB)_2 \cdot p\text{-}BQ]^{2-}$.

State	Energy [cm^{-1}]	Wavelength [nm]	fosc	T2 [au**2]	TX [au]	TY [au]	TZ [au]
1	25581.1	390.9	0.064642	0.8319	0.62964	0.64801	0.12466
2	25884.7	386.3	0.002082	0.02648	–0.14329	0.0768	0.00692
3	26596.2	376	0.058448	0.72348	0.59402	–0.59112	–0.14555
4	26615.5	375.7	0.043737	0.54099	0.4977	–0.54155	–0.00182
5	26857.5	372.3	0.035911	0.44019	–0.4475	0.48	0.0976
6	26874.9	372.1	0.159668	1.9559	1.00096	–0.97398	–0.07312

Supplementary Table 7. The absorption spectrum via transition electric dipole moments of $[DtBuCzB{\cdot}p\text{-}BQ]^{2-}$.

State	Energy [cm^{-1}]	Wavelength [nm]	fosc	T2 [au**2]	TX [au]	TY [au]	TZ [au]
1	16364	611.1	0.02084	0.41925	0.28161	–0.1796	0.5547
2	16851.4	593.4	0.016387	0.32015	–0.089	0.51961	–0.20551
3	20277.8	493.1	0.003141	0.05099	0.14311	0.06724	0.16122
4	21876.2	457.1	0.006433	0.09681	–0.20058	0.02613	–0.23642
5	24257.6	412.2	0.001245	0.0169	0.09279	–0.06058	0.06798
6	25504	392.1	0.002673	0.0345	–0.0435	–0.0205	–0.17942
7	25589.5	390.8	0.001351	0.01738	0.01436	0.1265	0.03423
8	26158.8	382.3	0.000267	0.00336	–0.04184	0.01741	0.03612
9	26998.6	370.4	0.096533	1.1771	0.07699	1.07643	0.11161
10	27610.2	362.2	0.030731	0.36642	0.58146	0.15331	–0.06942
11	27935.4	358	0.004372	0.05152	0.10589	–0.15706	0.12508
12	28860.2	346.5	0.016548	0.18877	–0.35303	–0.15801	0.19792
13	29157.7	343	0.116286	1.31296	–0.02519	1.13161	0.17827

Supplementary Table 8. The absorption spectrum via transition electric dipole moments of $[(DtBuCzB)_2 \cdot p\text{-BQ}]^{\bullet-}$.

State	Energy [cm^{-1}]	Wavelength [nm]	fosc	T2 [au**2]	TX [au]	TY [au]	TZ [au]
1	4967.9	2012.9	0.005239	0.34715	0.20878	0.54153	0.10152
2	5006.2	1997.5	0.00025	0.01641	0.08145	0.08802	0.0451
3	7082.9	1411.9	0.000042	0.00195	–0.039	0.01984	–0.00613
4	7210.3	1386.9	0.002263	0.10333	–0.04298	0.31613	0.03931
5	10774	928.2	0.001328	0.04057	0.07866	0.17331	0.06595
6	10804.1	925.6	0.003105	0.09463	0.18999	0.20283	0.13186
7	10969.6	911.6	0.00011	0.00331	0.0393	0.00543	0.04165
8	11047.5	905.2	0.002144	0.0639	–0.21477	0.12897	0.03379
9	15275	654.7	0.002722	0.05867	–0.1265	–0.18363	–0.09463
10	15450.3	647.2	0.011839	0.25226	–0.10325	–0.47434	–0.12883

Supplementary Table 9. The absorption spectrum via transition electric dipole moments of [DtBuCzB·*p*-BQ]$^{•−}$.

State	Energy [cm^{-1}]	Wavelength [nm]	fosc	T2 [au**2]	TX [au]	TY [au]	TZ [au]
1	10555.5	947.4	0.001846	0.05759	–0.07638	0.16213	–0.15958
2	12366.8	808.6	0.000324	0.00864	–0.01963	0.06523	–0.06322
3	13849	722.1	0.000007	0.00017	0.00843	0.00724	0.0071
4	16366.8	611	0.000665	0.01337	–0.08618	–0.0361	–0.06814
5	16410.5	609.4	0.000232	0.00466	–0.01683	0.04001	0.05264
6	19813	504.7	0.004356	0.07238	–0.13611	0.14016	–0.18496
7	21783.7	459.1	0.021139	0.31947	0.20382	–0.34585	0.39789
8	22879.8	437.1	0.001324	0.01906	–0.03746	0.00836	–0.1326
9	23629.3	423.2	0.066075	0.92059	0.46563	–0.25663	0.7987
10	23993	416.8	0.098637	1.35341	0.56525	–0.44401	0.91475

Supplementary Methods

Supplementary Method 1: Materials

Poly(methyl methacrylate) (PMMA), 4-Dimethylaminopyridine (DMAP), sodium phenate were purchased from Aladdin Chemicals, China. *p*-Benzoquinone (*p*-BQ), hydroquinone (H_2Q), 1,4-dutyrolactone, n-butyllithium and ferrocene were purchased from Energy Chemicals, China. 2-methoxy-1,4-benzoquinone ($BQ\text{-}OCH_3$) was purchased from TCI, China. DtBuCzB was obtained in collaboration with professor Chenglong Li (^{1}H-NMR was shown in Supplementary Fig. 16). Tetrabutylammonium hexafluorophosphate ($TBAPF_6$) was recrystallized for three times in anhydrous ethanol and dried under vacuum overnight before using. The indium tin oxide (ITO)-glass electrode was purchased from South China Xiang Science & Technology company.

Supplementary Method 2: Instrument characterization

UV-vis absorbance spectra and kinetic data were recorded using a Shimadzu UV-2600i PC double-beam spectrophotometer. Transient absorption spectra were obtained by femtosecond transient absorption spectrometer from Ultrafast Systems LLC. Fluorescence spectra and kinetic data were obtained with a Shimadzu spectrofluorimeter RF-5301PC. Cyclic voltammograms and differential pulse voltammograms were measured by Bio-logic electrochemical work station. The three-electrode cell, for cyclic voltammograms and differential pulse voltammograms measurement, consisted of a glass-carbon working electrode (3 mm dia., Chenhua, China), a Pt wire counter electrode (Chenhua, China) and an Ag wire reference electrode (Chenhua, China). Nuclear magnetic resonance spectra (NMR) were recorded with a ZhongkeNiujin AS 400 MHz NMR spectrometer, a BRUKER AVANCE 500 MHz NMR and a BRUKER AVANCE III 600 MHz NMR. Chemical shift values in H-NMR are given relative to TMS. The electron paramagnetic resonance (EPR) spectrum was obtained on a BRUKER E500 spectrometer. FT-IR spectra were measured on Vertex 80/80V FT-IR spectrometer over the range of 4000-400 cm^{-1} using a KBr plate. The calculations were performed at O3LYP/TZVP level by the ORCA 5.0.3 package using the conductor-like polarizable continuum model (CPCM).[S3-S6]

Supplementary Method 3: Electrochemistry

As shown in Supplementary Fig. 23, a three-electrode system was used for spectroelectrochemistry measurement, including a Pt net as the working electrode, a Pt wire as the counter electrode, and an Ag wire electrode as the reference electrode.

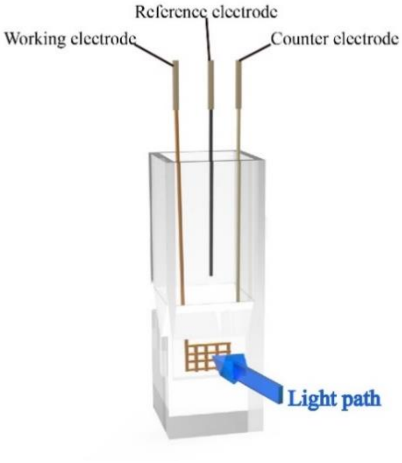

Supplementary Fig. 23. Diagram of electrochemical cell which was used to measure spectro-electrochemistry in solution ‘in situ’.

As shown in **Supplementary Fig. 24**, an H-type electrolytic cell including two-electrode (two Pt nets) was used for electroreduction of *p*-BQ. The preparation details can refer to our previous work.[S7] It could be described as below. The mixture of *p*-BQ (2.0×10^{-1} mol L^{-1}) and $TBAPF_6$ (1.0×10^{-1} mol L^{-1}) in CD_2Cl_2 was used as the working solution. The mixture of ferrocene (2.0×10^{-1} mol L^{-1}) and $TBAPF_6$ (1.0×10^{-1} mol L^{-1}) in CD_2Cl_2 was used as the counter solution to balance the charge. The reaction was stirred under –1.2 V for three hours in the glove box.

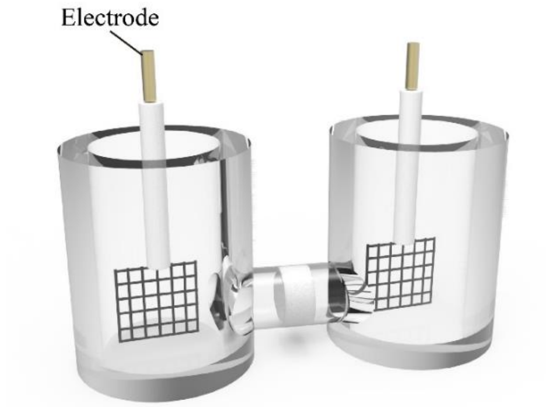

Supplementary Fig. 24. Diagram of electrolytic device.

Supplementary Method 4: Synthesis

Synthesis of hydroquinone di-lithium salt

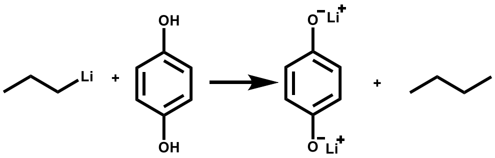

Supplementary Fig. 25. Synthetic route of hydroquinone di-anion.

A stock solution of 2 eq. n-butyllithium (2.5 mol/L in n-hexane) was added to a solution of hydroquinone (0.1 mol/L in THF) drop by drop under a nitrogen atmosphere at –78℃. After the reaction was completed, the solid was filtered, and the solid was repeatedly washed with anhydrous THF to remove the unreacted n-butyllithium, and the final product was obtained, 80% yield. ^{1}H-NMR and FT-IR spectra are shown in the Supplementary Fig. 17 and Fig. 20. ^{1}H NMR (400 MHz, THF-d_8) δ 6.49 (s, 4H).

Compared with the initial hydroquinone, the characteristic peak of the product disappeared at 7.5 ppm, indicating that the proton hydrogen of hydroquinone was robbed by the strong organic base (n-butyllithium). In FT-IR spectra, hydroquinone showed the characteristic peak at 3261 cm^{-1} and 1353 cm^{-1}, corresponding to the stretching vibration and bending vibration of –OH, respectively. Those peaks disappeared in hydroquinone di-lithium salt, which proved the deprotonation was complete.

Synthesis of PTMA-co-BP

PTMA-co-BP was prepared according to previous methods with some modifications.[S8, S9] It could be described as below:

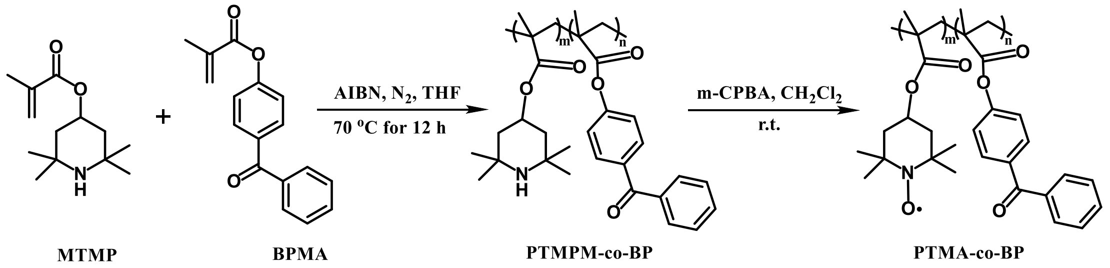

Supplementary Fig. 26. Synthetic route of PTMA-co-BP.

Synthesis of PTMPM-co-BP

2,2,6,6-tetramethylpiperidine methacrylate monomer (4.5 g, 20 mmol), benzoylphenyl methacrylate monomer (535 mg, 2 mmol) and azodiisobutyronitrile (AIBN) (20 mg, 0.12 mmol) were dissolved in 15 mL THF under nitrogen atmosphere. The system was stirred at 70 °C for 12 h. Then the product (PMTMP-co-BP)

was obtained by precipitating into ethyl acetate, filtered, and dried under vacuum, 60% yield. ^{1}H-NMR (400 MHz, Chloroform-*d*) was showed in Supplementary Fig. 18 and Fig. 19.

Synthesis of PTMA-co-BP

500 mg PMTMP-co-BP dissolved in 5 mL dichloromethane (CH_2Cl_2) was added into a 50 mL round-bottom flask. And 1 g meta-chloroperbenzoic acid (m-CPBA) dissolved in 15 mL CH_2Cl_2 was carefully added into the flask. The system was stirred for 10 min and washed with saturated sodium bicarbonate ($NaHCO_3$) solution for three times. Then, the organic phase was concentrated, precipitated in n-hexane at 0 ℃, filtered and dried to obtain the product (PTMA-co-BP), 80% yield. The structure was confirmed by FT-IR spectra (Supplementary Fig. 21) and the electron paramagnetic resonance spectrum (EPR) (Supplementary Fig. 22).

In FT-IR spectra, MTMP and BPMA showed the characteristic peak at 1634 cm^{-1}, corresponding to the C=C. This peak disappeared in PMTMP-co-BP, which proved the polymerization was complete. And the characteristic peak at 3400 cm^{-1} (corresponding to the N-H) disappeared, and the characteristic peak at 1362 cm^{-1} appeared in PTMA-co-BP, which proved the generation of the nitroxyl radical. The EPR spectrum of PTMA-co-BP is a broadened singlet signal, which is associated with the stable nitroxyl radical. (g value: 2.0074, Supplementary Fig. 22).

Supplementary Method 5: Fabrication of the electrofluorochromic (EFC) devices

Two-layer EFC devices

EFC solution: PMMA (24.7%, wt%), $TBAPF_6$ (1.1%, wt%), 1,4-dutyrolactone (73.8%, wt%), electro-Lewis base ($BQ\text{-}OCH_3$) (0.358%, wt%) and DtBuCzB (0.042%, wt%) in tetrahydrofuran (THF).

Ion storage solution: PTMA-co-BP (10 mg/mL) in THF.

As shown in Supplementary Fig. 27, first, the EFC film layer was deposited by drop coating on the first ITO glass in the glove box. Next, the ion storage film was deposited by spin coating on the second ITO glass (80 μL, 500 r.p.m., 30 s). Then, the ion storage layer was obtained from UV-crosslinking in the glove box (254 nm for 10 min). Finally, two-layer EFC device was fabricated by assembling the two ITO glasses together.

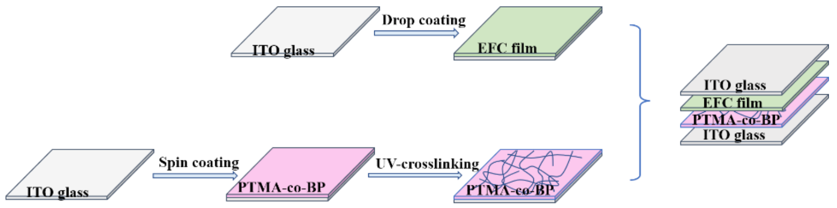

Supplementary Fig. 27. Fabrication process of two-layer EFC device.

Supplementary Method 6: The calculation of association constant (*K_a*)

Take DtBuCzB and *p*-BQ as an example:

$$\text{DtBuCzB} + \text{p-BQ} \underset{}{\overset{K_a}{\rightleftharpoons}} \text{DtBuCzB}\cdot\text{p-BQ}$$

$$K_a = \frac{[\text{DtBuCzB}\cdot p\text{−BQ}]}{[\text{DtBuCzB}][p\text{−BQ}]} \qquad \text{(Supplementary Equation 1)}$$

$$K_a = \frac{[\text{DtBuCzB}\cdot p\text{−BQ}]}{([\text{DtBuCzB}]_0 - [\text{DtBuCzB}\cdot p\text{−BQ}])([p\text{−BQ}]_0 - [\text{DtBuCzB}\cdot p\text{−BQ}])} \qquad \text{(Supplementary Equation 2)}$$

$$[\text{DtBuCzB}\cdot p - \text{BQ}] = \frac{\left([\text{DtBuCzB}]_0 + [p\text{−BQ}]_0 + \frac{1}{K_a}\right) - \sqrt{([\text{DtBuCzB}]_0 + [p\text{−BQ}]_0 + \frac{1}{K_a})^2 - 4[\text{DtBuCzB}]_0[p\text{−BQ}]_0}}{2} \qquad \text{(Supplementary Equation 3)}$$

Where [DtBuCzB·*p*-BQ] is the concentration of complex, $[\text{DtBuCzB}]_0$ is the concentration of the fix initial concentration of DtBuCzB, $[p\text{-BQ}]_0$ is the concentration of the initial concentration of *p*-BQ, [DtBuCzB] is the concentration of the DtBuCzB, when complexation have been dynamic equilibrium, [*p*-BQ] is the concentration of the *p*-BQ, when complexation have been dynamic equilibrium.

When the whole coordination process is dynamic, the relationship between fluorescence intensity changes and complex concentration as follows:

$$\text{Flu}_{\text{obs}} = \text{N}_{\text{DtBuCzB}} \times \text{Flu}_{\text{DtBuCzB}} \qquad \text{(Supplementary Equation 4)}$$

$$\text{N}_{\text{DtBuCzB}} = \frac{[\text{DtBuCzB}]}{[\text{DtBuCzB}] + [\text{DtBuCzB}\cdot p\text{−BQ}]} \qquad \text{(Supplementary Equation 5)}$$

$$\text{N}_{\text{DtBuCzB}\cdot\text{BQ}} = \frac{[\text{DtBuCzB}\cdot p\text{−BQ}]}{[\text{DtBuCzB}] + [\text{DtBuCzB}\cdot p\text{−BQ}]} \qquad \text{(Supplementary Equation 6)}$$

$$[\text{DtBuCzB}]_0 = [\text{DtBuCzB}] + [\text{DtBuCzB}\cdot p - \text{BQ}] \qquad \text{(Supplementary Equation 7)}$$

$$[p - \text{BQ}]_0 = [p - \text{BQ}] + [\text{DtBuCzB}\cdot p - \text{BQ}] \qquad \text{(Supplementary Equation 8)}$$

Put equation (Equation 5), (Equation 6), (Equation 7) and (Equation 8) into equation (Equation 4),

$$\frac{\text{Flu}_{[\text{DtBuCzB}]_0} - \text{Flu}_{\text{obs}}}{\text{Flu}_{[\text{DtBuCzB}]_0}} = \frac{[\text{DtBuCzB}\cdot p - \text{BQ}]}{[\text{DtBuCzB}]_0}$$

Where Flu_{obs} is the fluorescence intensity when complexation have been dynamic equilibrium, $\text{Flu}_{[\text{DtBuCzB}]0}$ is the initial fluorescence intensity of DtBuCzB, $\text{N}_{[\text{DtBuCzB}]}$ is the molar percentage content of DtBuCzB, $\text{N}_{[\text{DtBuCzB}\cdot p\text{-BQ}]}$ is the molar percentage content of [DtBuCzB·*p*-BQ].

Based on the above discussion, the association constant (K_a) of DtBuCzB with *p*-BQ (the stoichiometry of DtBuCzB·*p*-BQ is 1:1 ratio) is expressed as follow:

$$\frac{\mathrm{Flu}_{[\mathrm{DtBuCzB}]_0}-\mathrm{Flu}_{\mathrm{obs}}}{\mathrm{Flu}_{[\mathrm{DtBuCzB}]_0}}=\frac{\left([\mathrm{DtBuCzB}]_0+[p-\mathrm{BQ}]_0+\frac{1}{\mathrm{K_a}}\right)-\sqrt{\left([\mathrm{DtBuCzB}]_0+[p-\mathrm{BQ}]_0+\frac{1}{\mathrm{K_a}}\right)^2-4[\mathrm{DtBuCzB}]_0[p-\mathrm{BQ}]_0}}{2\times[\mathrm{DtBuCzB}]_0} \quad \text{(Supplementary Equation 9)}$$

Note: The calculation methods of association constant K_a value for DtBuCzB and *p*-BQ after electrochemical stimulation was the same as above.

Supplementary Notes

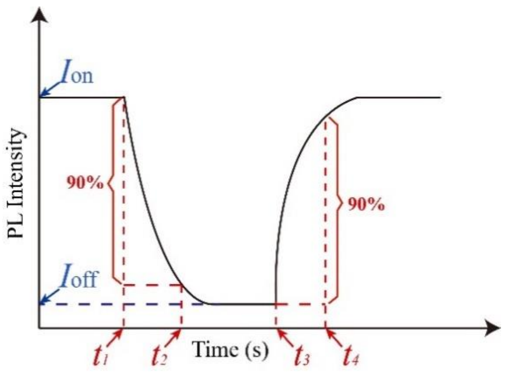

Supplementary Fig. 28. Diagram of fluorescence change under the voltage stimulation.

The fluorescence quenching efficiency (η, %), calculated at λ_{max} by Equation 10:

$$\eta = \frac{I_{\mathrm{on}} - I_{\mathrm{off}}}{I_{\mathrm{on}}} \times 100\% \qquad \text{(Supplementary Equation 10)}$$

The switching time, which is the time at a 90% of the full fluorescence change occurs after applying potential. It can be calculated by Equation 11 or Equation 12:

$$t_{\mathrm{on}} = t_2 - t_1 \qquad \text{(Supplementary Equation 11)}$$

$$t_{\mathrm{off}} = t_4 - t_3 \qquad \text{(Supplementary Equation 12)}$$

The maximum contrast ratio (CR), calculated at λ_{max} by Equation 13:

$$CR = {I_{on}}/{I_{off}} \qquad \text{(Supplementary Equation 13)}$$

Supplementary References

1. Kasprzak, A., Borys, K. M., Molchanov, S. & Adamczyk-Woźniak, A. Spectroscopic insight into supramolecular assemblies of boric acid derivatives and β-cyclodextrin. *Carbohydr. Polym.* **198**, 294-301 (2018).
2. Rudlof, J. *et al.* Synthesis of bifunctional boron-lewis acids – thorough investigation of the adduct formation with pyrimidine. *Eur. J. Inorg. Chem.* **2022**, 202100842 (2022).
3. Helmich-Paris, B., de Souza, B., Neese, F. & Izsák, R. An improved chain of spheres for exchange algorithm. *J. Chem. Phys.* **155**, 104109 (2021).
4. Izsák, R. & Neese, F. An overlap fitted chain of spheres exchange method. *J. Chem. Phys.* **135**, 144105 (2011).
5. Izsák, R., Neese, F. & Klopper, W. Robust fitting techniques in the chain of spheres approximation to the Fock exchange: The role of the complementary space. *J. Chem. Phys.* **139**, 094111 (2013).
6. Neese, F., Wennmohs, F., Hansen, A. & Becker, U. Efficient, approximate and parallel Hartree–Fock and hybrid DFT calculations. A 'chain-of-spheres' algorithm for the Hartree–Fock exchange. *Chem. Phys.* **356**, 98-109 (2009).
7. Yang, G. *et al.* A Multiple Chirality Switching Device for Spatial Light Modulators. *Angew. Chem. Int. Ed.* **60**, 2018-2023 (2020).
8. He, J. *et al.* Highly transparent crosslinkable radical copolymer thin film as the ion storage layer in organic electrochromic devices. *ACS Appl. Mater. Interfaces* **10**, 18956-18963 (2018).
9. Gu, C. *et al.* A strategy of stabilization via active energy-exchange for bistable electrochromic displays. *CCS Chem.* **4**, 2757-2767 (2022).
