# Supplementary material for: An electrochemically responsive B–O dynamic bond to switch photoluminescence of boron-nitrogen-doped polyaromatics
Source: Nat Commun. 2024 Jun 17;15:5166. doi: 10.1038/s41467-024-48918-6 (PMC11183244; doi:10.1038/s41467-024-48918-6)
Supplement: Supplementary file 3 — Description of Additional Supplementary Files [file 41467_2024_48918_MOESM3_ESM.pdf]

Description of Additional Supplementary Files

File Name: Supplementary Data 1
Description: Cartesian coordinates (O3LYP) for DtBuCzB (S_0)

File Name: Supplementary Data 2
Description: Cartesian coordinates (O3LYP) for [DtBuCzB·p-BQ]$^{2-}$ (S_0)

File Name: Supplementary Data 3
Description: Cartesian coordinates (O3LYP) for [(DtBuCzB)2·p-BQ]$^{2-}$ (S_0)
